# Workup for Suspected Brugada Syndrome: Two Case Reports for the General Practitioner

**DOI:** 10.7759/cureus.21921

**Published:** 2022-02-05

**Authors:** Michael Blotner, Omkar Betageri, William Miles, Kun Xiang

**Affiliations:** 1 Internal Medicine, University of Florida College of Medicine, Gainesville, USA; 2 Cardiology, University of Florida College of Medicine, Gainesville, USA

**Keywords:** ventricular arrhythmia, brugada electrocardiogram pattern, brugada phenocopy, procainamide, st-elevation, brugada syndrome, brugada

## Abstract

While a large proportion of ST-segment elevation on EKG is related to myocardial ischemia, the differential diagnosis must include pericarditis, channelopathies, and various genetic conditions. Identifying and working up such abnormalities present a challenge to primary care providers (PCPs). We present two clinical cases of young male patients with ST-segment elevation in anteroseptal leads suspicious for Brugada syndrome and show how to risk stratify and manage them.

Our first case presents a 23-year-old male with no past medical history with acute onset substernal chest pain, shortness of breath, and palpitations. Initial workup revealed negative serial troponins and normal B-type natriuretic peptide (BNP). The EKG revealed ST elevation in lead V2. An evaluation for Brugada syndrome was pursued. Upon completion of a procainamide challenge, it was determined that he did not have Brugada syndrome and was shortly discharged.

Our second case presents a 33-year-old male with no pertinent cardiac medical history who presented to an outpatient cardiology clinic after discovering an incidental ST elevation in V2 on EKG. His family history was negative for early atherosclerotic cardiovascular events or sudden cardiac death. The patient’s initial workup was negative. Suspicion for Brugada syndrome leads to performing a procainamide challenge, which was significant for ST changes in the anterolateral leads. He was asymptomatic during the challenge and initial presentation, and no further intervention was indicated. He was advised to avoid sodium channel blocking medications and treat any fevers and was sent for genetic testing.

These cases illustrate the importance of maintaining an appropriate suspicion for Brugada syndrome in young patients with minimal ischemic risk factors. We discuss a guideline-directed algorithmic workup for PCPs in suspicious individuals. Stratifying patients based on the presence of symptoms, history of tachyarrhythmias, and EKG findings before and after drug challenge allows physicians to guide further management of these patients.

## Introduction

One of the most common diagnostic tools in both the hospital and ambulatory setting for general practitioners is the use of an EKG. ST-segment elevation on EKG can increase suspicion for myocardial infarction (MI), but other causes may include pericarditis, early repolarization, left ventricular hypertrophy, left bundle branch block, ventricular aneurysm, or takotsubo cardiomyopathy [1,2]. One additional cause of ST-segment elevation is Brugada syndrome, which is associated with ventricular arrhythmia and sudden cardiac death [3-6]. EKGs in patients with Brugada syndrome have a characteristic ST-segment elevation in the anterolateral leads (V1 to V3) [4,6-8]. It poses a challenge to primary care clinicians to assess the risks of sudden cardiac death in such patients. Here, we present two clinical cases of patients with ST-segment elevation in anteroseptal leads and provide fundamental suggestions on risk stratification and management.

## Case presentation

Case 1

**Figure 1:**
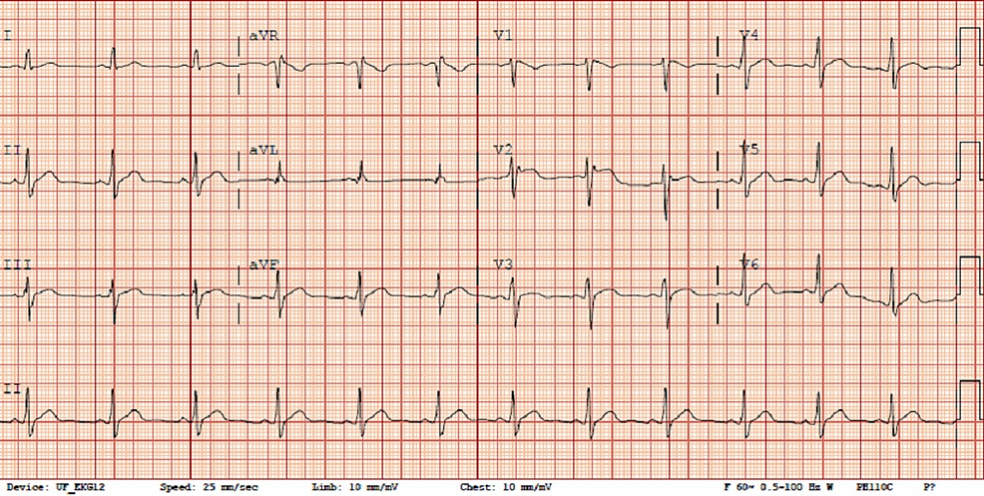
Baseline EKG in the emergency department.

A 23-year-old male with no significant past medical or family history presented to the emergency department for acute onset substernal chest pain, shortness of breath, and palpitations that occurred 30 minutes after putting out a small kitchen fire amidst consumption of alcohol. When emergency medical service (EMS) arrived at the scene, the patient was diaphoretic and in severe respiratory distress. On arrival at the emergency department, his symptoms had resolved. Initial workup was significant for negative serial troponins, B-type natriuretic peptide (BNP) within normal limits, and lactic acid of 5.2 mmol/L. EKG obtained is shown in Figure 1.

**Figure 2:**
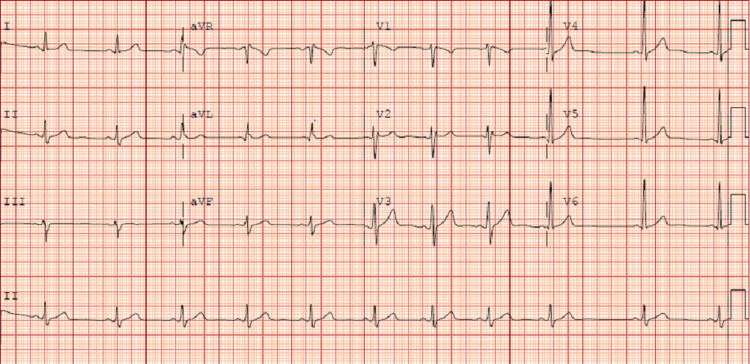
EKG obtained after procainamide challenge.

The patient was then admitted to the hospital’s cardiology service for further workup and management. During the admission, there was a concern for Brugada syndrome, given the morphology of the above EKG in V2. Echocardiogram performed showed no abnormalities. The patient was taken for an IV procainamide challenge. The resulting EKG after a full 1,000 mg challenge is shown in Figure 2.

Given the patient’s unchanged EKG with a pharmacologic challenge along with atypical symptoms for Brugada syndrome, the patient was discharged home without further workup or management.

Case 2

**Figure 3:**
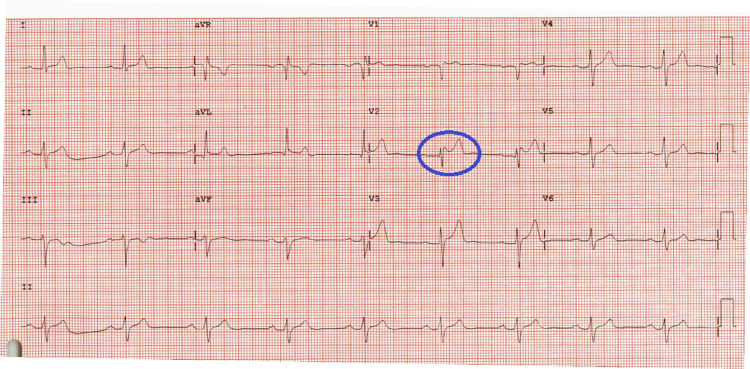
Baseline EKG showing Brugada pattern type 2 with a saddleback appearance in V2.

A 33-year-old male with a past medical history of attention deficit hyperactivity disorder (ADHD) was referred to our cardiology office for an abnormal EKG before refilling his methylphenidate prescription. He reports one occurrence of panic attacks in the past year, where he had palpitations and diaphoresis before an important exam. He denies any history of syncope or pre-syncope. Family history is notable for a father with a 4.8 cm ascending aortic aneurysm and a paternal uncle that died from an aortic dissection. There is no family history of sudden death or syncope. He previously had a 24-hour Holter monitor and echocardiogram that were unremarkable. Overall, the patient felt well and denied any chest pain, shortness of breath, palpitations, or orthopnea. Referral EKG is shown in Figure 3.

**Figure 4:**
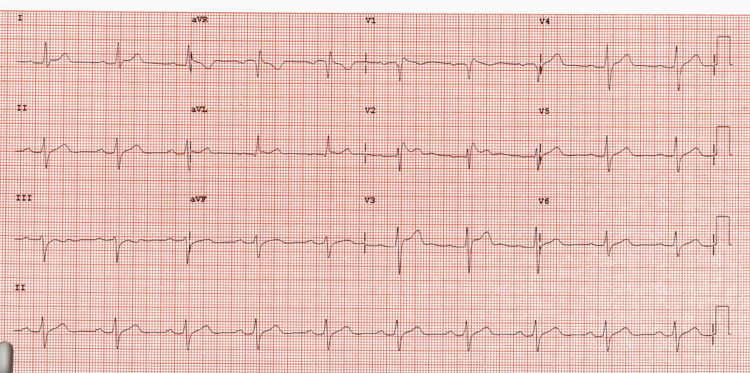
EKG at minute 14 of procainamide infusion (700 mg) showing the conversion from Brugada pattern type 2 to type 1.

**Figure 5:**
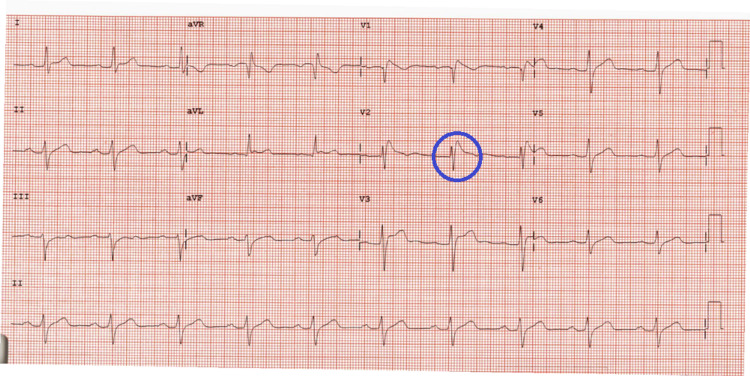
EKG at minute 20 of procainamide (1,000 mg) showing Brugada pattern type 1 with coved ST-segment elevation in V1-V2.

Given the concern for Brugada syndrome based on the findings in V1-V2, the patient underwent a procainamide challenge with the following EKGs shown in Figures 4, 5.

The above EKGs show the conversion of the type 2 Brugada pattern to the type 1 Brugada pattern. This confirms the diagnosis of Brugada syndrome. Due to the patient’s lack of family history or personal history of syncope or tachyarrhythmia, no further workup or intervention was indicated. However, genetic tests were sent and the patient was advised that first-degree relatives should obtain screening EKGs. He was also advised to avoid drugs with sodium channel blocking activity and was made aware that fever can exacerbate arrhythmias in Brugada syndrome.

## Discussion

Brugada syndrome is an autosomal dominant disorder with a variable expression that presents with abnormal EKG findings in conjunction with an increased incidence of ventricular tachyarrhythmia and sudden cardiac death [9]. The clinical disease has a male predominance, with an average age of diagnosis at 41 years old [10,11]. Primary care providers (PCPs) serve a vital role in the prevention of cardiac disease. In both the adult and pediatric domains, it is recommended that PCPs focus cardiac screening on risk factors and family history of any cardiac abnormalities [6,12,13]. The American Academy of Pediatrics specifically recommends screening for family history of sudden cardiac death or sudden cardiac arrest for primary prevention [6]. In particular, the PCP is especially critical in the context of Brugada syndrome, as multiple family members within their patient panels may be affected. Current recommendations include screening all first-degree relatives of any patient diagnosed with Brugada syndrome [9].

Our cases serve as fundamental examples of the workup algorithm of suspected Brugada syndrome, which may be encountered by the PCP. Both cases presented young men with no prior cardiac history with EKG findings consistent with Brugada pattern type 2. Prompt and accurate diagnosis of Brugada syndrome is critical, as symptomatic patients have an elevated risk of sudden cardiac death from arrhythmia [5]. While the prevalence of Brugada syndrome in patients with Brugada pattern EKG is not well established, some studies estimate a 10% event rate at 2.5 years [3].

**Figure 6:**
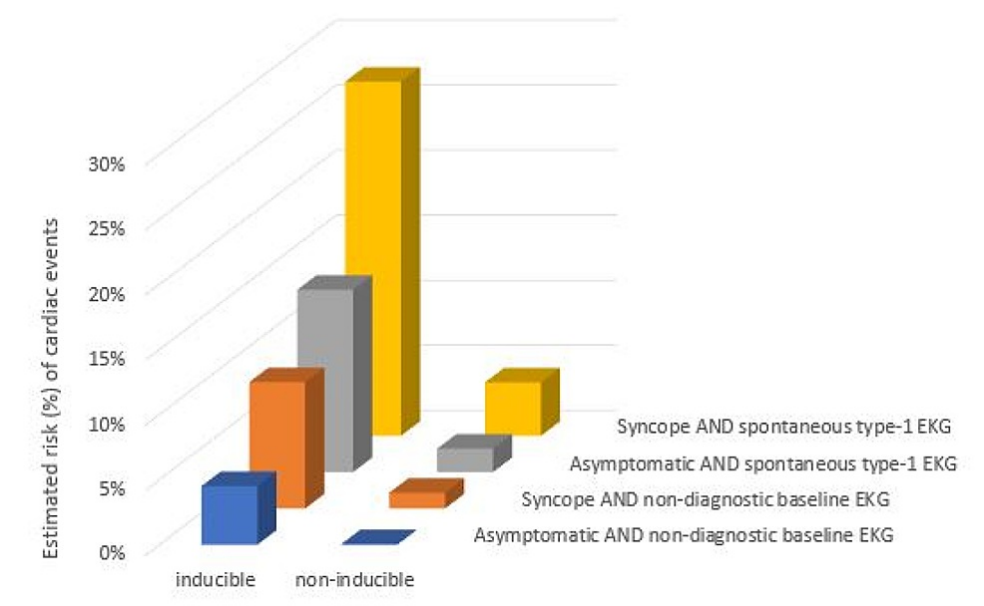
Probability of cardiac events during follow-up estimated by logistic regression analysis, according to the presence of symptoms, inducibility of arrhythmias, and type of baseline ECG. Adapted from [5,7].

In case 1, the morphology of the ST segment continues to have the saddleback appearance of a type 2 pattern throughout the procainamide challenge and does not change. This is classified as a negative pharmacological challenge, and therefore no further workup was indicated for the patient. In contrast, case 2 was noted to have a conversion from the type 2 to type 1 Brugada pattern with the procainamide challenge. No ventricular arrhythmia was induced. Although case 2 confirmed a diagnosis of Brugada syndrome, an absence of a cardiac arrest, tachyarrhythmia, or syncope in his history indicates that he does not require further workup. This is based on stratification data, which demonstrate that an inducible type 1 pattern in the absence of prior symptoms or inducible arrhythmia has an elevated but overall low chance of developing a ventricular arrhythmia (Figure 6) [5,7]. However, given that he does have a positive diagnosis, he was evaluated for genetic components of Brugada syndrome, and his first-degree relatives were advised to seek evaluation for Brugada syndrome. He was also advised to avoid medications that inhibit cardiac sodium channels (class I antiarrhythmics, tricyclic antidepressants (TCAs), and lithium) in the future, as this would risk inducing an arrhythmia. Another consideration in Brugada syndrome patients is prompt treatment of any fevers with antipyretics for arrhythmia prevention [14].

**Figure 7:**
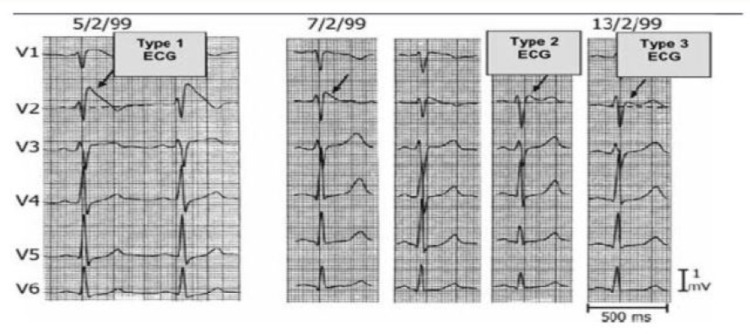
Brugada syndrome. Examples of type 1, type 2, and type 3 Brugada patterns. Obtained and permission granted for reuse from [4].

There are three different types of Brugada EKG patterns: type 1, type 2, and type 3 (Figure 7). They can be distinguished by analyzing the ST segments in the V1-V2 precordial leads. In type 1, the ST segment is described as an elevated coved pattern with a high takeoff point (2 mm elevation from baseline) with no identifiable r’, which is followed by a negative and symmetric T-wave. In type 2, the ST segment is noted to have a saddleback appearance, with a J-point 2 mm elevated from baseline and the terminal portion of the ST segment 1 mm above baseline. Type 3 is similar to type 2, except the ST segment is less than 1 mm above the baseline. In patients where Brugada syndrome is suspected without corresponding EKG, it may be possible to elicit these patterns by moving the right precordial leads superiorly to the second, third, or fourth intercostal space, as this has shown to increase the sensitivity of detecting the EKG pattern [8,15,16]. The basis for this observation is the current theory that tissue and microscopic changes located in the right ventricular outflow tract are responsible for the syndrome [7]. The presence of a type 1 EKG pattern in conjunction with symptoms concerning for ventricular tachyarrhythmia or syncope is sufficient to make the diagnosis of Brugada syndrome. For type 2 EKG patterns, further workup is necessary to confirm the diagnosis.

**Figure 8:**
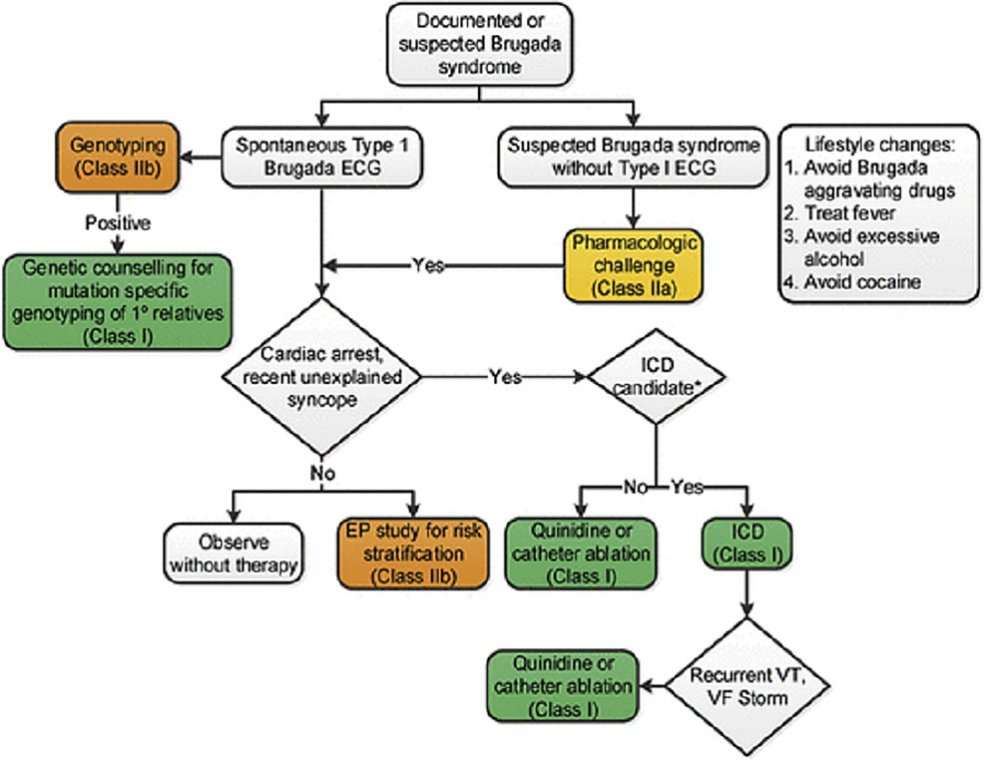
Suspected Brugada syndrome. Guideline-directed workup and primary prevention. Obtained and permission granted for reuse from [9]. ICD, implantable cardioverter-defibrillator; EP, electrophysiology; VT, ventricular tachycardia; VF, ventricular fibrillation.

Patients with a type 2 EKG finding and clinical presentation concerning for tachyarrhythmia should undergo a drug challenge with a sodium channel blocker such as flecainide or procainamide (Figure 8) [8,16]. Many Brugada syndrome patients with family history components have genetic abnormalities in cardiac sodium channels that result in a decreased influx of sodium into the myocyte. Administration of a sodium channel blocker attempts to mimic this genetic abnormality. If the pharmacologic challenge elicits a type 1 EKG pattern in patients with cardiac arrest or unexplained syncope, current guidelines recommend implantable cardioverter-defibrillator (ICD) placement as first-line treatment (Class I recommendation) [9,14]. This would target the prevention of death from ventricular tachyarrhythmia, the major source of sudden death in Brugada syndrome patients. In patients where ICD placement is contraindicated or has failed, medical management with either quinidine or amiodarone can be added to the regimen [17,18]. If antiarrhythmic therapy fails, then catheter ablation can be considered [19]. For all patients presenting with type 1 EKG findings, genotyping including the SCN5A gene is recommended (Class IIb recommendation).

## Conclusions

These cases illustrate the importance of maintaining an appropriate suspicion for Brugada syndrome in young patients with minimal ischemic risk factors. We discussed a guideline-directed algorithmic workup in suspicious individuals. Stratifying patients based on the presence of symptoms, history of tachyarrhythmias, and EKG findings before and after drug challenge allows physicians to guide further management of these patients.
